# Mechanistic insights into the synergistic interactions between macrolides and beta-lactams in bacterial infections

**DOI:** 10.3389/fcimb.2026.1894766

**Published:** 2026-10-15

**Authors:** Qiaochun Lu, Xubo Qian, Yandie Li, Yao Liu, Zijin Chen, Le He, Yilin Yu, Zhigang Jin, Yuting Shi

**Affiliations:** 1 Department of Pediatrics, Affiliated Jinhua Hospital, Zhejiang University School of Medicine, Jinhua, China; 2 Department of Pediatrics, Jinhua Maternal and Child Health Care Hospital, Jinhua, China; 3 Department of Rheumatology Immunology and Allergy, Children’s Hospital, Zhejiang University School of Medicine, Hangzhou, Zhejiang, China; 4 China National Clinical Research Center of Respiratory Diseases, Respiratory Department, Beijing Children’s Hospital, Capital Medical University, National Center for Children’s Health, Beijing, China; 5 Division of Pediatric Pulmonology, Chengdu Women’s and Children’s Central Hospital, School of Medicine, University of Electronic Science and Technology of China, Chengdu, China; 6 Department of Pediatrics, Jinhua Hospital of TCM Affiliated to Zhejiang University of Traditional Chinese Medicine, Jinhua, China; 7 Jinhua Municipal Central Hospital, Shaoxing University, Jinhua, China; 8 College of Life Sciences, Zhejiang Normal University, Jinhua, China

**Keywords:** antibacterial mechanisms, community-acquired pneumonia, immune modulation, macrolide antibiotics, pharmacokinetics, resistance, synergy, beta-lactam antibiotics

## Abstract

β-Lactams and macrolides are two cornerstone classes of antimicrobials in clinical practice. Traditionally, this combination was considered potentially antagonistic. However, accumulating evidence from basic and clinical research has begun to challenge this view and indicates that the two classes may exert complementary and synergistic effects in specific clinical contexts. This review first describes the mechanisms of action and resistance of both antibiotic classes, and then presents clinical evidence supporting the use of combination therapy. The greatest clinical benefit of combination therapy is observed in adults with severe community-acquired pneumonia (CAP). By contrast, results from non-severe CAP or pediatric populations have been inconsistent. We then systematically review potential synergistic mechanisms, including complementary antibacterial targets, macrolide penetration facilitated by β-lactam-induced cell-wall injury, suppression of bacterial repair and stress responses, biofilm disruption, immunomodulation, and quorum-sensing interference. We also classify the strength of evidence for each proposed mechanism. Finally, we compare the pharmacokinetic/pharmacodynamic (PK/PD) profiles of azithromycin, clarithromycin, and erythromycin and assess their potential impact on combination efficacy. We also discuss safety concerns, including adverse effects, drug interactions, and selection for resistance, along with strategies to address these issues. It should be emphasized that most mechanistic evidence derives from *in vitro* or animal studies, and only a minority comes from clinical research. The combination should be used with strict attention to indications, and its benefits should not be extrapolated indiscriminately to other conditions. We conclude that β-lactam–macrolide combination therapy demonstrates promising synergy in severe CAP and other specific infections. The multi-level mechanistic framework lends biological plausibility to the observed synergy, but biological synergy does not guarantee clinical benefit in all patients. Only when used in the right patients, at the right time, and with the right drug combination can this therapeutic strategy produce clinical benefit.

## Graphical Abstract

**Figure:**
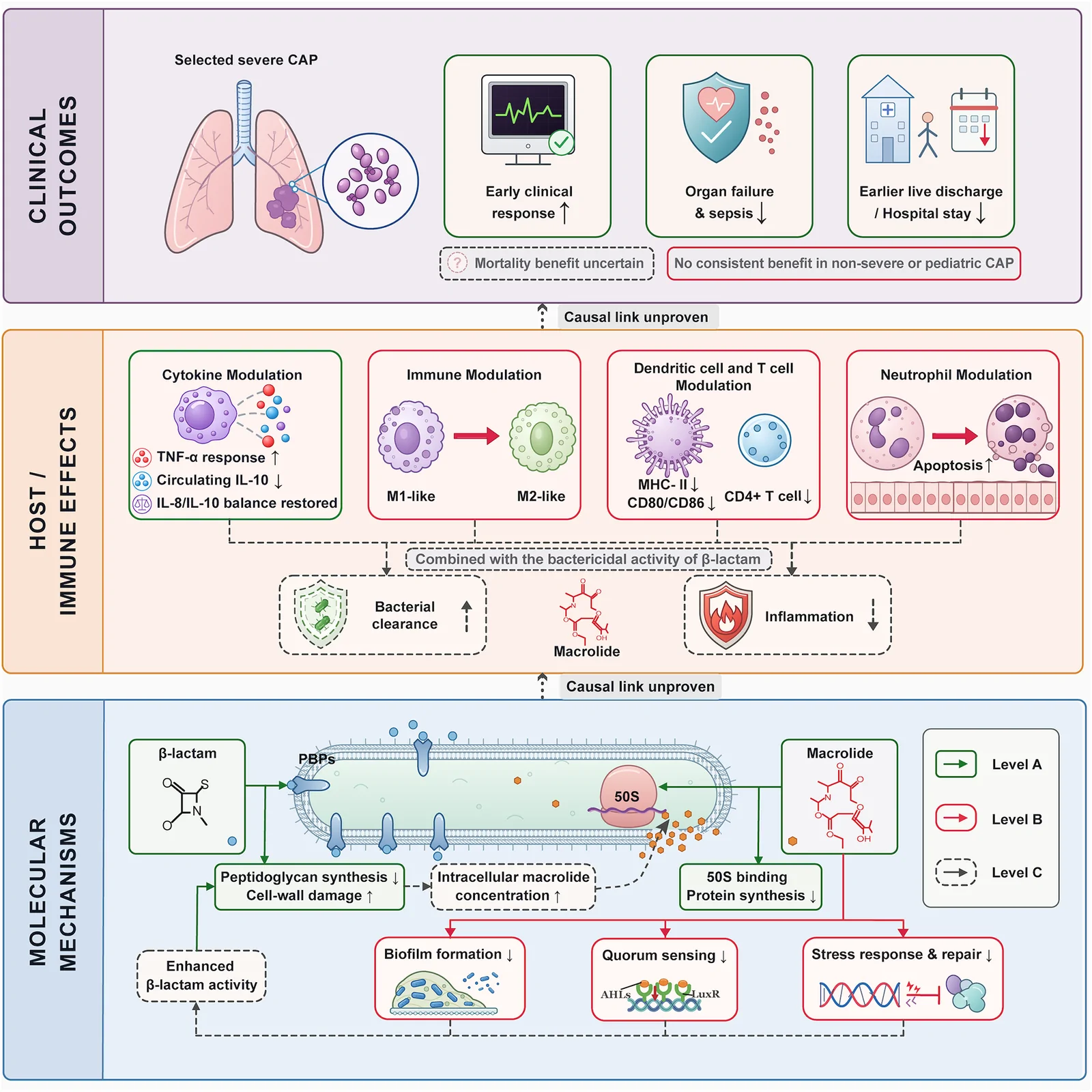
Multilevel interactions between macrolides and β-lactams. At the molecular level, β-lactam-mediated cell-wall damage may facilitate macrolide entry into bacteria, whereas macrolides may enhance β-lactam activity by inhibiting biofilm formation and quorum sensing and by interfering with bacterial stress responses. At the immune level, the immunomodulatory effects of macrolides, together with the bactericidal activity of β-lactams, may promote bacterial clearance and reduce inflammation. In selected patients with severe community-acquired pneumonia (CAP), these combined effects may improve early clinical response, reduce organ failure and sepsis, and shorten hospital stay. A mortality benefit remains uncertain, particularly in non-severe or pediatric CAP. Solid green, solid red, and dashed black lines indicate evidence Levels A, B, and C, respectively (see Section 2, “Interpretation of evidence”).

## 1 Introduction

β-lactam antibiotics (β-lactams) and macrolide antibiotics (macrolides) are among the most commonly used antimicrobial classes in clinical practice and play a crucial role in treating bacterial infections. β-lactams, including penicillins and cephalosporins, offer potent broad-spectrum activity, relatively low systemic toxicity, established clinical efficacy, and a wide range of indications (Lima et al., 2020; Turner et al., 2022). Macrolides such as erythromycin, clarithromycin and azithromycin are often used for their activity against atypical pathogens and for their anti-inflammatory properties (Pradhan et al., 2024; Taguchi et al., 2024).

Macrolides act by inhibiting bacterial protein synthesis, thereby producing a bacteriostatic state. Historically, it was hypothesized that they might antagonize the bactericidal activity of β-lactams, which led to general hesitancy about their concomitant use (Drago et al., 2011). However, recent data suggest that their use in combination may improve overall antibacterial activity and reduce mortality in specific patient populations (Giamarellos-Bourboulis et al., 2024; Wang et al., 2025), and a meta-analysis showed significantly better outcomes in patients with severe community-acquired pneumonia (CAP) who received combination treatment rather than β-lactam monotherapy (Wang et al., 2025). These data emphasize the need to re-evaluate traditional views on the combination and to understand the mechanisms underlying their synergistic effects.

In recent years, the mechanisms through which macrolides enhance the activity of β-lactams have received much attention. It has been demonstrated that macrolides have various biological activities that may synergize with β-lactams, such as inhibition of bacterial protein synthesis, modulation of inflammatory responses, and disruption of biofilm formation (Giamarellos-Bourboulis et al., 2024). These effects can result in improved bacterial clearance and reduced inflammation in the host, ultimately leading to better clinical outcomes (Giamarellos-Bourboulis et al., 2024; Prizao et al., 2025; Wang et al., 2025). These findings underscore the clinical potential of this combination and identify opportunities for further studies aimed at optimizing antibiotic use in the context of the continued rise of antimicrobial resistance.

Against this background, this review aims to evaluate the clinical and mechanistic evidence for β-lactam-macrolide combination therapy and to clarify when this combination offers true clinical advantage. We first describe the mechanisms of action and resistance of both drug classes, and then present the clinical evidence supporting combination therapy. We next synthesize the mechanistic evidence to explain how the combination may improve clinical outcomes, and finally discuss PK/PD considerations, safety concerns, and antimicrobial stewardship, together with strategies to address these issues.

## 2 Interpretation of evidence

We used a single three-level framework to grade both clinical evidence and mechanistic claims. Level A: the claim is supported by high-quality human evidence, such as systematic reviews, meta-analyses of randomized controlled trials (RCTs), or well-designed large RCTs. For mechanistic claims, Level A requires consistent, direct experimental evidence from clinically relevant systems, including human-derived data; for claims limited to established molecular pharmacological facts, such as distinct drug targets or known binding sites, Level A may be assigned without requiring human-derived data. Level B: the claim is supported by well-conducted non-randomized human studies, RCTs with important methodological limitations, or consistent preclinical evidence from animal, ex vivo, or *in vitro* studies. Level C: the claim is supported only by preliminary, indirect, or hypothesis-generating evidence, or it lacks direct evidence of clinical translation. This three-level framework reflects the strength and certainty of the evidence and distinguishes established synergy from probable or hypothesized mechanisms.

The potential efficacy of β-lactam–macrolide combination therapy may be constrained by heterogeneity across studies, including variations in infection type, disease severity, pathogen distribution, treatment regimen, and methodological design. Given this substantial clinical and methodological diversity, we synthesize the evidence narratively rather than deriving a pooled effect estimate.

## 3 Mechanisms of action and resistance in β-lactams

### 3.1 Structure, classification, and mechanism of action

β-lactams are an important class of antimicrobials characterized by a β-lactam ring that is responsible for antibacterial activity. This ring enables these agents to inhibit bacterial cell-wall synthesis, causing cell lysis and death. The class includes penicillins, cephalosporins, other β-lactams and β-lactamase inhibitors (Lima et al., 2020; Turner et al., 2022).

The penicillins (e.g., penicillin G, methicillin, amoxicillin, piperacillin, and pivmecillinam) were the first β-lactams to be discovered. They act well against many Gram-positive organisms and some Gram-negative species. Cephalosporins, which are structurally related to penicillins, are further divided into five generations, with representatives such as cefradine, cefuroxime, ceftriaxone, cefpirome, and cefazolin. Other β-lactams include carbapenems, cephamycins, oxacephems, and monobactams, which have distinct features and clinical indications. Carbapenems (e.g., imipenem and meropenem) have an extremely wide spectrum of activity and are not readily hydrolyzed by β-lactamases. They are generally reserved for serious infections and are an important option for treating multidrug-resistant bacterial infections. Monobactams such as aztreonam are predominantly active against Gram-negative organisms and are generally well tolerated in patients with penicillin allergy.

The structural diversity in each class of β-lactams accounts for the differences in effectiveness against various bacteria as well as their sensitivity to β-lactamases, which are produced by certain bacteria to confer resistance. Understanding the structure-function relationships of these antibiotics is essential for developing novel agents and strategies against antibiotic resistance, which remains an important clinical challenge (Lima et al., 2020; Turner et al., 2022; Harris et al., 2024). Furthermore, differences among β-lactam agents in tissue penetration, protein binding, and elimination routes directly influence their efficacy at specific infection sites (e.g., lung parenchyma, cerebrospinal fluid) and the selection of partner agents in combination regimens. These pharmacokinetic properties, together with pathogen susceptibility profiles, should be carefully weighed in clinical decision-making.

Penicillin-binding proteins (PBPs) are essential bacterial enzymes that catalyze the final steps in the bacterial cell wall biosynthesis by crosslinking the peptidoglycan layer, thereby maintaining structural integrity and mechanical rigidity of the cell (Carlson et al., 2025). Penicillin and other β-lactams bind to these proteins and inhibit their activity, interrupting cell-wall biosynthesis and eventually causing bacterial lysis. In addition to their main action on PBPs, β-lactam antibiotics have other mechanisms. For example, ceftazidime, cefotaxime, ceftriaxone, and imipenem are associated with altered autolytic activity in bacteria (Krawczyk-Balska and Lipiak, 2013; Dorr, 2021). Cefoxitin can competitively interact with β-lactamases and reduce hydrolysis of other β-lactams (Culić et al., 2002). At sub-MIC concentrations, piperacillin inhibits biofilm matrix production and weakens structural integrity (Fonseca et al., 2004). These observations broaden the pharmacology of β-lactams, but their relevance depends on the agent, pathogen, and exposure.

### 3.2 Mechanisms of β-lactam resistance

The mechanisms for bacterial resistance against β-lactams are complex but mainly include the expression of certain proteins and enzymes that counteract the effect of those antibiotics (Dorr, 2021) (**Figure 1**). The production of β-lactamases is one of the most important mechanisms, since these enzymes hydrolyze the β-lactam ring, thereby inactivating the antibiotic. There are multiple classes of β-lactamases such as extended-spectrum β-lactamases and carbapenemases, which can degrade a broad spectrum of β-lactams. The presence of these enzymes is associated with excessive β-lactam use and confers a selective advantage that allows resistant bacteria to thrive (Kim et al., 2021). β-lactamases are usually encoded on mobile genetic elements such as plasmids, enabling horizontal gene transfer between bacterial populations and exacerbating resistance (Sun et al., 2019; Castaneda-Barba et al., 2024). β-lactamases can also shelter otherwise susceptible bacteria within the same environment, contributing to treatment failure in polymicrobial infections (Mora-Ochomogo et al., 2025).

**Figure 1:**
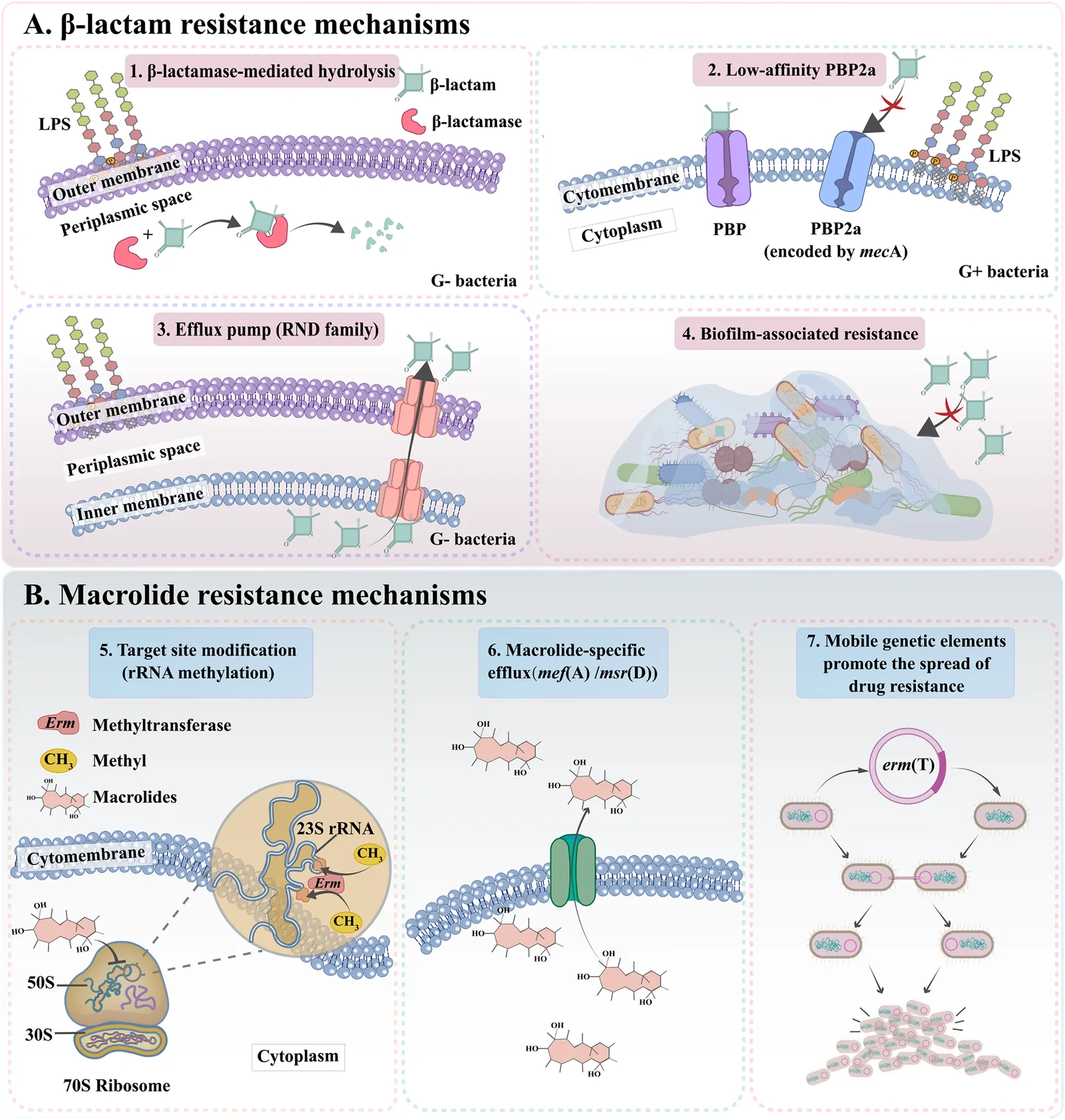
Mechanisms of resistance to β-lactam and macrolide antibiotics. **(A)** β-lactam resistance: 1. β-Lactamases hydrolyze the β-lactam ring and inactivate the drug; 2. *mecA*-encoded PBP2a has low binding affinity for β-lactams, allowing peptidoglycan synthesis and cell-wall integrity to be maintained; 3. RND-family efflux pumps actively export antibiotics and reduce intracellular concentrations; 4. Biofilms restrict antibiotic penetration and sustain resistance. **(B)** Macrolide resistance: 5. *erm*-encoded 23S rRNA methyltransferases impede macrolide binding to the 50S ribosomal subunit; 6. *mef(A)*/*msr(D)*-encoded macrolide-specific efflux systems actively export the drug; 7. Resistance genes, including *erm(T)*, spread on mobile genetic elements and promote horizontal dissemination of antimicrobial resistance.

In addition to β-lactamase production, expression of penicillin-binding protein 2a (PBP2a), an important β-lactam target, is a major resistance mechanism. Compared with standard PBPs, PBP2a has low affinity for β-lactams, allowing methicillin-resistant *Staphylococcus aureus* (MRSA) to continue synthesizing its cell wall despite β-lactam exposure (Adedeji-Olulana et al., 2024). PBP2a is often encoded by the *mecA* gene, which can be transferred between bacteria and facilitate the spread of resistance (Peacock and Paterson, 2015; Castaneda-Barba et al., 2024).

Another resistance mechanism involves actively pumping the antibiotic out of the bacterial cell (Glen and Lamont, 2021; Vareschi et al., 2025). The efflux pumps, particularly from the resistance-nodulation-division (RND) superfamily, mediate the active extrusion of β-lactams outside the cell, thus lowering their intracellular concentrations, as well as their antimicrobial efficacy (Dulanto Chiang and Dekker, 2024). This process is especially important in Gram-negative bacteria, whose outer membrane provides an additional barrier to antibiotic entry. The interaction between efflux pumps and outer-membrane permeability is a major determinant of the overall resistance phenotype in these pathogens (Krishnamoorthy et al., 2017).

Reduced outer-membrane permeability is an established resistance mechanism in Gram-negative bacteria (Mammeri et al., 2025). The outer membrane of Gram-negative organisms is inherently impermeable to many hydrophilic compounds. β-lactams pass the barrier mostly via water-filled protein channels called porins, such as OmpF and OmpC in *Enterobacteriaceae*. The main mechanisms are downregulation, loss, or altered function of porins (Su et al., 2025), which reduces antibiotic influx and prevents the drug from reaching intracellular concentrations needed to inhibit PBPs. A clinically relevant example is carbapenem resistance in *Klebsiella pneumoniae*, which is often associated with loss of OmpK35/OmpK36 porins and production of a β-lactamase. This illustrates how porin deficiency can combine with enzymatic hydrolysis to confer high-level resistance (Lee et al., 2024; Su et al., 2025).

Biofilm formation is another escape mechanism used by microbial communities in response to close interactions among embedded microbes or environmental threats. Biofilms are attached microbial communities enclosed in a protective extracellular polymeric substance (EPS) matrix (Jandl et al., 2024). Biofilm-mediated resistance to β-lactams involves several intertwined mechanisms (Jandl et al., 2024; Nasrollahian et al., 2024). The EPS matrix limits the penetration of antibiotics and may sequester β-lactamases, which results in localized enzyme inactivation. Within biofilms, nutrient and oxygen gradients slow bacterial growth and induce metabolic dormancy, reducing the effectiveness of β-lactam antibiotics targeting active peptidoglycan synthesis. This heterogeneous environment can also promote persister formation and activate stress responses that modify cell-wall physiology and autolytic activity. In addition, biofilms facilitate horizontal gene transfer, increasing the rate at which bacteria acquire or share resistance determinants such as β-lactamase genes (Jandl et al., 2024; Nasrollahian et al., 2024).

It should also be noted that other factors, such as pH, may affect the development and expression of bacterial resistance. For example, studies have shown that low pH can increase resistance of *Klebsiella pneumoniae* to β-lactams by inducing PBPs with low susceptibility to β-lactam inhibition (Beagle and Levin, 2026). Porin permeability is also dynamically regulated by periplasmic H^+^ and K^+^ concentrations (Cano Muniz et al., 2025). Bacterial physiology, which is influenced by the surrounding environment, may therefore affect resistance potential.

## 4 Mechanisms of action and resistance in macrolides

### 4.1 Structure, classification, and mechanism of action

Macrolides are an important group of antibacterials defined by a macrocyclic lactone ring that is required for biological activity. The most well-known members of this class include erythromycin, azithromycin, and clarithromycin, each distinguished by their specific structural features and pharmacological properties. Erythromycin, the first macrolide discovered, is a 14-membered-ring macrolide used mainly to treat respiratory tract infections. Azithromycin is a 15-membered-ring macrolide with a prolonged half-life, improved tissue penetration, activity against a wider range of organisms including atypical bacteria, and weaker CYP3A4 inhibition. Clarithromycin, another 14-membered macrolide, is a derivative of erythromycin with improved acid stability and bioavailability, which improves its effectiveness in treating a variety of bacterial infections (Dinos, 2017; Pradhan et al., 2024).

The main action mode of macrolides is their inhibitory effect on bacterial protein synthesis through binding to the 23S rRNA part of the 50S ribosomal subunit, thus blocking the peptidyl transferase center and inhibiting the extension of the nascent polypeptide chain in translation (Vazquez-Laslop and Mankin, 2018). In some cases, this effect may also be bactericidal, especially when the macrolide remains bound to the ribosome for a prolonged period (Svetlov et al., 2020). Differences in ring structure and side chains not only produce clinically relevant variations in pharmacokinetics, drug interactions, and tolerability, but also determine marked discrepancies in immunomodulatory activity, anti-virulence effects, and the strength of available clinical evidence (Taguchi et al., 2024).

Structural variations also profoundly influence safety profiles. The 14-membered ring and side-chain substituents of erythromycin and clarithromycin confer higher CYP3A4 affinity, rendering them more prone to drug interactions than azithromycin. All macrolides can prolong the QT interval via hERG potassium channel blockade, although reported incidences vary, likely reflecting differences in tissue accumulation and metabolite activity. The motilin-like prokinetic effect, which is unique to erythromycin, accounts for its greater gastrointestinal intolerance than the other two agents. Appreciation of these structure–safety associations is a prerequisite for rational macrolide selection in combination regimens, with further clinical considerations provided in Section 7.3.

### 4.2 Mechanisms of macrolide resistance

The emergence of macrolide resistance, especially in clinical settings, is one major problem because of the extensive therapeutic use of these agents and the rapid selection and dissemination of resistant strains (Kalizang'oma et al., 2025). The primary resistance mechanisms against macrolides include target site modification, active efflux, and enzymatic inactivation (Goltermann et al., 2024; Xu et al., 2025b) (**Figure 1**). The most common mechanism is target-site modification, in which bacteria acquire *erm* genes encoding methyltransferases, a family of enzymes that methylate 23S rRNA in the 50S ribosomal subunit (Alexander et al., 2025; Graham et al., 2026). Such resistance is usually mediated by *erm*-type methyltransferases, which methylate the rRNA and remodel the drug-binding pocket so that high-affinity drug-ribosome interaction is abolished (Svetlov et al., 2021). This methylation process greatly decreases the affinity of macrolides for the ribosome, leading to high-level resistance. In addition to rRNA modification, efflux pumps such as *mef(A)* and *msr(D)* further increase macrolide resistance by actively pumping the drug out of bacterial cells, thereby lowering intracellular antibiotic concentrations (Southon et al., 2020). Less frequently, bacteria may produce enzymes to inactivate the macrolide, including esterases encoded by ere genes or phosphotransferases encoded by mph genes, which chemically alter the drug and make it ineffective (Zielinski et al., 2021; Singh et al., 2025).

In addition, it has been demonstrated that the resistance mechanisms can vary among different bacterial species: for example, in *M. pneumoniae*, resistance is often linked to point mutation of the 23S rRNA conferring high level resistance to macrolides, complicating treatment options (Kim et al., 2022a; Wang et al., 2023). Resistance has increased especially in *Staphylococcus aureus*, where spread of *erm*-mediated resistance has limited the effectiveness of conventional macrolides such as erythromycin (Miklasinska-Majdanik, 2021). The emergence of macrolide resistance in *M. pneumoniae* also indicates that further studies are needed to monitor this trend and develop new treatment options (Leng et al., 2024).

Many macrolide-resistant isolates carry mobile genetic elements that enable resistance genes to be transferred horizontally between species (Dechene-Tempier et al., 2021). For instance, *erm(T)*, which is found in pathogens such as *Glaesserella parasuis*, contributes to the spread of resistance (Zhang et al., 2025). This genetic plasticity promotes the spread of resistance genes and complicates antimicrobial therapy by conferring cross-resistance to structurally related antibiotics.

Moreover, macrolide resistance has serious clinical consequences due to treatment failure or persistent infections. In *Pseudomonas aeruginosa*, prolonged exposure to macrolides has been associated with ribosomal mutations and adaptive resistance mechanisms (Goltermann et al., 2022). This highlights the need to understand all resistance mechanisms, including mutation and efflux-pump expression, when designing therapeutic approaches.

## 5 Clinical evidence for β-lactam-macrolide combination therapy

Given that the strength and nature of the evidence vary considerably across different clinical contexts, we present the following data separately by infection type and pathogen category.

### 5.1 Combination therapy for CAP

Community-acquired pneumonia (CAP) is the clinical setting in which the combined use of macrolides and β-lactams has been most extensively studied. **Table 1** lists the key clinical studies for the application of combined treatment in patients with CAP. Since the effect of combination therapy is influenced by the severity of the patient’s disease, we will separately discuss these two different situations: severe CAP (defined by CURB-65 ≥ 3 or requiring ICU admission) and non-severe CAP (outpatient or general ward management).

**Table 1:** Key clinical studies supporting or questioning β-lactam–macrolide combination therapy.

Study (author, year, ref)	Study design	Population/comparison	Sample size	Key findings	Key limitations	Level of evidence
Giamarellos-Bourboulis et al., 2024	Double-blind RCT	Adults hospitalized with CAP; clarithromycin + β-lactam vs placebo + β-lactam	N = 278 (139 per arm)	Improved day-4 composite endpoint (≥50% symptom reduction + ≥30% SOFA decrease or ≥80% PCT decrease or PCT <0.25 ng/mL): 68% vs. 38% (OR 3.40, P < 0.0001). Fewer organ failure: HR 0.30 (P = 0.0041). Fewer sepsis events: HR 0.49 (P = 0.029). Shorter time to discharge alive: median 10 vs. 12 days (P < 0.0001). No significant increase in SAEs.	Single macrolide, single regimen; composite primary endpoint, not powered for mortality; modest sample size limits precision of secondary outcomes	A
Prizao et al., 2025	meta-analysis of RCTs	CAP; macrolide add-on therapy vs β-lactam monotherapy	7 RCTs; total N = 1,517	Higher estimated clinical success: RR 1.17 (95% CI not reported); Mortality estimates limited by small event numbers and heterogeneous severity.	Heterogeneous severity, regimens, outcomes, and trial eras; limited precision for mortality	A
Postma et al., 2015	cluster-randomized crossover trial, CAP-START	Adults with CAP on non-ICU wards; strategy favoring β-lactam monotherapy vs β-lactam + macrolide or fluoroquinolone strategies	2,283 patients in 4 strategies; 656 received β-lactam monotherapy vs 585 combination	β-Lactam monotherapy strategy was noninferior to β-lactam + macrolide for 90-day mortality	Open-label strategy trial; performance bias possible; low atypical pathogen prevalence limits generalizability; not powered for subgroup analyses	B
Garin et al., 2014	open-label RCT	Moderately severe hospitalized CAP; β-lactam monotherapy vs β-lactam + macrolide	580 patients (290 per arm)	Noninferiority of monotherapy not established overall (wide CIs); differences concentrated in patients with atypical pathogens or more severe illness	Open-label design; noninferiority margin may have been too narrow; subgroup findings underpowered; overall CIs wide	B
Williams et al., 2017	prospective cohort	Children hospitalized with CAP; β-lactam monotherapy vs empirical macrolide combination therapy	2,536 children (1,299 monotherapy; 1,237 combination)	No routine outcome benefit with combination therapy in children; Length of stay, treatment failure, and readmission rates not significantly different.	Nonrandomized pediatric cohort; residual confounding by severity and indication (sicker children more likely to receive macrolides); incomplete pathogen classification	B
Wang et al., 2025	network meta-analysis	Severe CAP; comparison of multiple empirical regimens	35 studies (19 RCTs, 16 observational); total N not pooled	β-Lactam + macrolide ranked favorably for mortality and clinical success among empirical strategies	Predominantly observational; indirect comparisons; residual confounding by severity and treatment timing; heterogeneity in study eras and populations	C

#### 5.1.1 Severe CAP

The rationale for adding a macrolide to a β-lactam in severe CAP rests on a hierarchy of evidence that converges on early clinical benefit, but diverges on mortality (Giamarellos-Bourboulis et al., 2024; Prizao et al., 2025; Wang et al., 2025) (**Figure 2**). In the double-blind ACCESS trial, adjunctive clarithromycin significantly improved a composite early clinical and inflammatory endpoint and was associated with fewer organ-failure and sepsis events and a shorter time to discharge alive (Giamarellos-Bourboulis et al., 2024). This high-quality trial provides direct, class-specific support for early improvement, though it was not powered for mortality.

**Figure 2:**
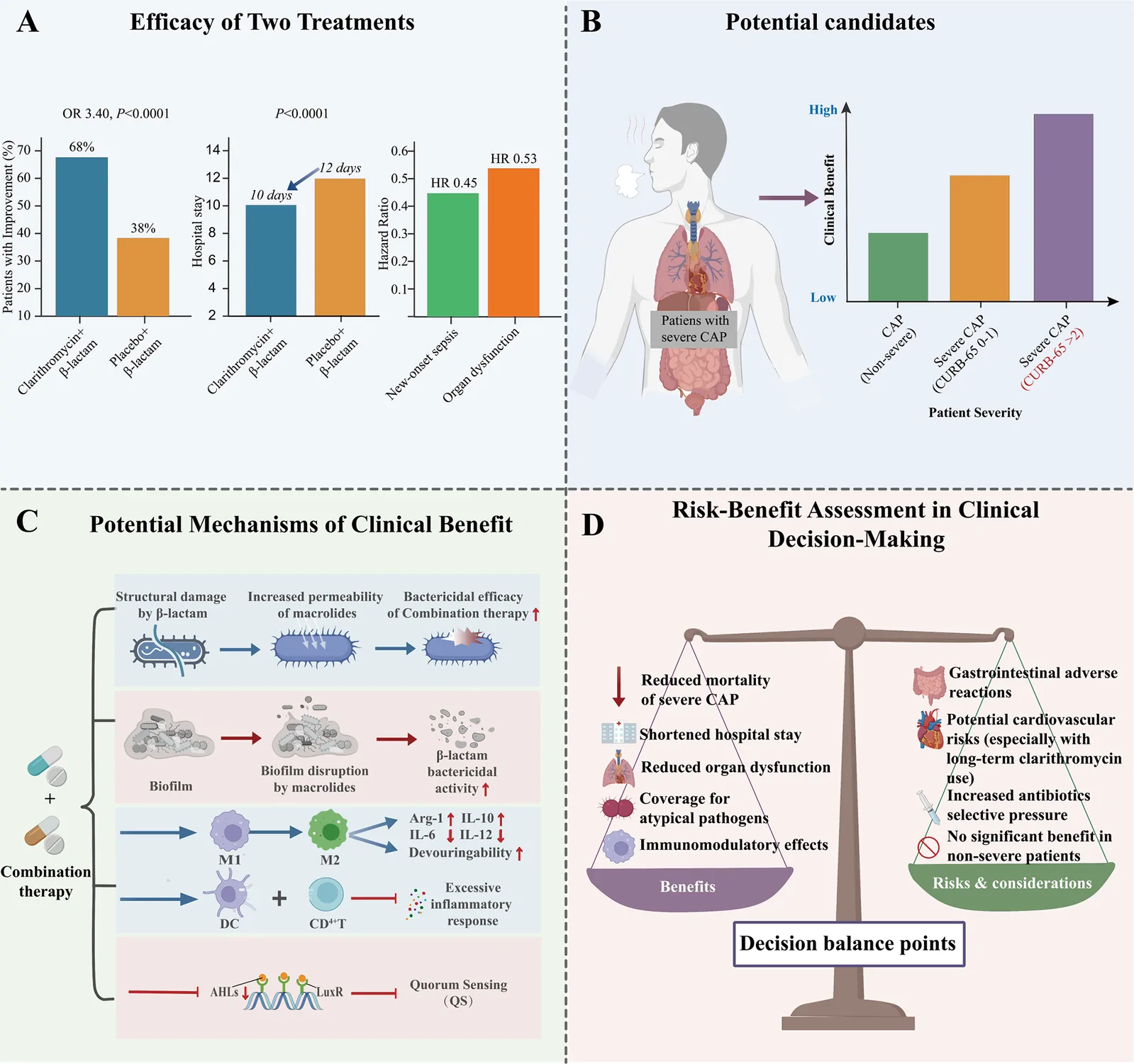
Clinical evidence, candidate populations, proposed mechanisms, and benefit-risk assessment of β-lactam-macrolide therapy for community-acquired pneumonia (CAP). **(A)** In ACCESS, adding clarithromycin to a β-lactam improved an early composite clinical and inflammatory endpoint. Certainty regarding a mortality benefit is lower because that signal comes mainly from observational or indirect comparisons. **(B)** Benefit is most plausible in selected patients with severe CAP; current evidence does not support routine use in non-severe adult or pediatric CAP. **(C)** Complementary spectrum and several bacterial or host-directed pathways may contribute, but penetration, biofilm, quorum-sensing, efflux, stress-response, and immunomodulatory mechanisms have not been shown to mediate clinical benefit causally. **(D)** Clinical decisions should balance severity and the probability of an atypical pathogen against toxicity, drug interactions, selection for resistance, and opportunities for reassessment and de-escalation.

Supporting this, meta-analyses and observational studies have suggested greater clinical success and lower mortality in hospital patients, with the strongest signal in severe CAP (Ceccato et al., 2019; Ito et al., 2021; Kim et al., 2022b; Bai et al., 2024; Prizao et al., 2025; Wang et al., 2025). However, these estimates are derived predominantly from non-randomized comparisons and indirect treatment rankings, leaving them vulnerable to confounding by indication, illness severity, and treatment timing. The network meta-analysis by Wang et al. (Wang et al., 2025), for instance, ranked the combination favorably for mortality, but its point estimate relies heavily on indirect comparisons across heterogeneous populations.

Taken together, the evidence supports a probable benefit in selected patients with severe CAP. Early clinical improvement is well established, whereas a mortality benefit remains suggestive rather than definitive and should not be assumed for every macrolide or patient.

#### 5.1.2 Non-severe and pediatric CAP

In non-severe and pediatric populations, the evidence does not support routine macrolide addition. Multiple studies, including the cluster-randomized CAP-START trial and an open-label RCT in moderately severe CAP, have failed to demonstrate consistent superiority of combination therapy over β-lactam monotherapy, with any potential advantage largely confined to patients with atypical pathogens or more severe illness (Garin et al., 2014; Postma et al., 2015; Bai et al., 2024). Pediatric cohort data do not show consistent routine benefit from empirical macrolide combination therapy in unselected children; reported benefits are mainly observed in school-aged children or when atypical pathogens are suspected (Ambroggio et al., 2012; Leyenaar et al., 2014; Ambroggio et al., 2016; Williams et al., 2017; Al Saeedy et al., 2020).

These findings, combined with the well-documented risks of gastrointestinal adverse effects, QT prolongation, drug interactions, and resistance selection (Fillatre et al., 2025), argue against extrapolating the positive signals from severe CAP to low-risk adults or children. The inconsistency across severity strata likely reflects differences in pathogen distribution, comorbidity burden, and study design, but the overall conclusion is clear: the early clinical improvement observed in severe disease does not generalize to lower-risk populations.

#### 5.1.3 Current guideline positions

Guidelines consistently stratify recommendations by disease severity, though the strength of their endorsement varies. The 2019 ATS/IDSA guideline supports β-lactam–macrolide combination for hospitalized and severe CAP, based largely on observational data and expert consensus (Metlay et al., 2019). In contrast, the 2023 ERS/ESICM/ESCMID/ALAT guideline, applying GRADE methodology, conditionally favors adding a macrolide over a fluoroquinolone in severe CAP but rates the evidence as very low certainty (Martin-Loeches et al., 2023). The 2026 ATS update, which addresses treatment duration rather than regimen selection, recommends shorter courses for non-severe cases and at least 5 days for severe CAP, without altering the 2019 regimen recommendations (Jones et al., 2026).

This variation reflects both the evolving evidence base and differing grading methodologies. Collectively, however, the guidelines converge on a single principle: any potential benefit of macrolide addition is most pronounced in severe illness or confirmed atypical pathogens, and routine use in low-risk populations is unsupported.

### 5.2 Combined application in other respiratory infections

Combined antibiotic therapy, especially the combination of macrolides and β-lactams, has been explored in several respiratory infections, including chronic bacterial bronchitis, bronchiectasis, whooping cough, invasive pneumococcal infection, and sinusitis. The potential value of the combination differs across these conditions and may reflect broader antimicrobial coverage, host-directed effects, or pathogen-specific mechanisms.

#### 5.2.1 Chronic bacterial bronchitis and bronchiectasis

In addition to the treatment of CAP, macrolides and β-lactam antibiotics have been studied in chronic bacterial bronchitis and bronchiectasis, both conditions being associated with chronic bacterial infection and airway inflammation. Macrolides have anti-inflammatory effects that may be beneficial in patients with bronchiectasis. Long-term macrolide therapy (azithromycin) appears to be associated with a reduced frequency of exacerbations in chronic respiratory diseases such as chronic obstructive pulmonary disease or bronchiectasis, mainly via anti-inflammatory and immunomodulatory effects (Chalmers et al., 2019; Lundgren, 2025). Although these studies primarily evaluated macrolide maintenance therapy rather than acute co-administration with a β-lactam, the host-directed effects of macrolides provide a biological rationale for adjunctive use when β-lactams are required for acute bacterial exacerbations. Direct randomized evidence for acute β-lactam–macrolide combination treatment remains limited.

#### 5.2.2 Pertussis

Macrolides remain first-line therapy for macrolide-susceptible *Bordetella pertussis*. In one study, 125 *B. pertussis* isolates obtained from patients treated with monotherapy using a single antibiotic showed a high incidence of macrolide resistance (62.4% [78/125]); fourteen days after treatment, bacterial clearance was higher in β-lactam-treated patients (68.8%, 44/64) than in macrolide-treated patients (50.8%, 31/61) (χ² = 4.18, P = 0.04). Within macrolide-susceptible isolates, clearance at day 7 was higher in the macrolide group than in the β-lactam group (χ² = 4.49, P = 0.03), whereas macrolide-resistant isolates had lower clearance rates. These results indicate that the relative contribution of each drug class varies with the resistance phenotype. Certain β-lactams, such as piperacillin, may represent alternative antibacterial options for macrolide-resistant *B. pertussis* (Mi et al., 2021). Because this study compared monotherapies, the clinical benefit of concurrent β-lactam–macrolide treatment was not directly assessed.

#### 5.2.3 Invasive pneumococcal infection

Pneumonia and meningitis caused by invasive pneumococci are settings in which combination therapy may have clinical value. Because of increasing resistance of *S. pneumoniae* to antibiotics, combination approaches have been explored. Studies suggest that adding a macrolide to a β-lactam may improve outcomes in patients with severe pneumococcal infection (Martínez et al., 2003; Prizao et al., 2025; Wang et al., 2025). The potential benefit may extend beyond direct antibacterial activity and include broader empirical coverage and host-directed immunomodulation. Most available evidence, however, is derived from observational studies or analyses of severe CAP rather than pathogen-specific randomized trials, and high-level macrolide resistance may limit direct anti-pneumococcal activity. The clinical value of the combination is therefore likely to vary with disease severity and pathogen susceptibility.

#### 5.2.4 Chronic rhinosinusitis and biofilm-associated infection

Given the documented presence of bacterial biofilms in patients with refractory chronic rhinosinusitis (Zahedi et al., 2025) and the reported biofilm-disrupting properties of macrolides (Lees et al., 2020; Yamabe et al., 2020), combination antimicrobial therapy may be mechanistically complementary in this population. By modifying the biofilm and inflammatory microenvironment, macrolides may facilitate β-lactam activity against susceptible, actively growing bacteria. Clinical studies of macrolides in chronic rhinosinusitis have generally evaluated long-term low-dose therapy rather than acute combination treatment; therefore, the additional clinical benefit of β-lactam–macrolide therapy in biofilm-associated rhinosinusitis remains to be established.

Across these respiratory infections, the contribution of macrolides appears to differ by pathogen and clinical setting. In atypical infections, the principal value is broader antimicrobial coverage, whereas in biofilm-associated or highly inflammatory infections, host-modulatory and anti-virulence effects may provide additional complementarity to β-lactam activity. These differences may partly account for the variable clinical benefit of β-lactam–macrolide therapy outside CAP.

### 5.3 Evidence discrepancies among different macrolides in combination therapy

As detailed in Section 4.1, azithromycin, clarithromycin, and erythromycin differ fundamentally in structure, pharmacokinetic profiles, and safety characteristics. These differences are reflected in an uneven distribution of clinical evidence for their use in combination with β-lactams; the agents should not be considered interchangeable in clinical practice.

With respect to high-level clinical evidence for combination therapy, clarithromycin is supported by the only direct randomized controlled trial (ACCESS) (Giamarellos-Bourboulis et al., 2024), which demonstrated improved early clinical composite endpoints and reduced new organ failure as an adjunct to β-lactams in severe CAP; however, the trial was not powered for mortality, and its findings cannot be extrapolated to other macrolides. Azithromycin’s combination evidence derives mainly from preclinical studies, including observed efflux modulation (Sugimura et al., 2008), anti-biofilm (Hoffmann et al., 2007), and quorum-sensing (Molinari et al., 1993; Zeng et al., 2016; Apostolaki et al., 2024) in *P. aeruginosa* models. However, no RCT has shown that these effects translate into reduced mortality or severity when azithromycin is combined with β-lactams. Its use in CAP combination regimens is primarily driven by atypical pathogen coverage rather than proven synergistic bactericidal benefit. The general CAP evidence should not be assumed to provide high-level support for azithromycin-specific synergy. Erythromycin contributes virtually no contemporary evidence on acute CAP combination therapy; existing data are largely confined to long-term monotherapy for chronic airway inflammation and are not relevant to acute combination regimens.

Furthermore, although all three agents are subject to resistance mechanisms mediated by ribosomal methylation (*erm*) or efflux pumps (*mef*/*msr*), β-lactam co-administration does not reliably reverse any of these phenotypes, and resistance prevalence as well as clinical breakpoints vary across agents and pathogens. Therefore, when appraising the indications for combination therapy, the specific macrolide used in each study must be strictly distinguished, and positive conclusions derived from a single agent should not justify the extension of combination regimens to other members of the same class. The direct evidence sources and major evidentiary gaps for each agent in combination therapy are summarized in **Table 2**.

**Table 2:** Agent-specific clinical evidence for β-lactam–macrolide combination therapy.

Macrolide agent	Direct evidence for β-lactam combination (source & context)	Major evidentiary gaps for combination indication
Clarithromycin	Highest-level human evidence (ACCESS RCT): confirmed early clinical improvement and reduced organ failure as adjunct to β-lactam in severe CAP	Mortality benefits not established (RCT not powered); results not extrapolatable to azithromycin or erythromycin
Azithromycin	Preclinical only (*in vitro*/animal models): anti-biofilm, quorum-sensing interference, immunomodulation in *P. aeruginosa*; CAP use based on atypical coverage, not proven synergy	No high-grade RCT for mortality or severity outcomes when combined with β-lactams; experimental findings species-specific and clinically unvalidated
Erythromycin	No contemporary evidence for acute CAP combination; data from long-term monotherapy in chronic airway disease (anti-inflammatory, not synergy)	No RCT or observational cohort testing β-lactam + erythromycin in acute severe infection; tolerability/interaction burdens preclude acute empirical use

### 5.4 Safety evidence for combination therapy

Safety must be considered as a critical dimension when evaluating combination regimens that may offer complementary or synergistic activity. Although recent data suggest improved antibacterial responses with macrolide–β-lactam treatment, the combination may introduce additional adverse drug reactions (ADRs). Large pharmacoepidemiologic cohort studies (including over 100,000 patients) have shown that macrolide therapy is associated not only with more gastrointestinal adverse events but also with an elevated risk of cardiovascular death and a modest increase in myocardial infarction (MI) risk (Wu et al., 2023). In that study, azithromycin was associated with a moderate but statistically significant increase in MI, whereas β-lactam and doxycycline prescriptions were not linked to greater cardiovascular risk. A pharmacogenetic analysis further found that certain polymorphisms predicted adverse reactions such as abdominal pain and diarrhea, particularly during azithromycin or erythromycin treatment (Skryabina et al., 2024), suggesting that genetic screening may eventually help identify patients at greater risk and inform drug selection.

However, most studies have focused on efficacy, and the safety evidence for combination therapy remains comparatively limited. The only relevant randomized double-blind controlled trial (ACCESS) in adults with CAP found no increase in adverse events during treatment (Giamarellos-Bourboulis et al., 2024); nevertheless, the trial was not powered for rare adverse events and excluded patients with significant baseline QTc prolongation or arrhythmias, limiting its generalizability to higher-risk populations.

It should be explicitly noted that the safety data for β-lactam–macrolide combination therapy are notably less robust than the efficacy data. The cardiovascular risk signal derives primarily from large pharmacoepidemiologic studies, which, despite their large sample sizes, rely on administrative databases and are subject to potential confounding (e.g., underlying cardiac disease and other risk factors). Pharmacogenetic studies remain preliminary, with small sample sizes and no prospective validation, and their clinical utility remains uncertain. Taken together, the evidence suggests that the incremental risk of adding a macrolide may be acceptable in severe CAP but must be weighed carefully in non-severe settings, where the efficacy benefit is marginal or absent (**Figure 2**).

## 6 Mechanism of interaction

The effect of β-lactam–macrolide combination therapy is governed by multiple interrelated determinants, including drug exposure levels, target engagement efficiency, intrinsic pathogen characteristics, and host immune status. To exert interactive actions—encompassing inhibition of cell-wall synthesis and protein translation, modulation of bacterial stress responses, interference with biofilm formation, disruption of quorum sensing, and remodeling of host immunity—both agents must reach the infection site within an overlapping therapeutic window and maintain effective concentrations. Synergy at the mechanistic level can reasonably be inferred only when the combined effect exceeds the sum of the independent activities of each agent and extends beyond mere complementary spectrum coverage.

It should be explicitly emphasized that the present section (Section 6) is devoted to a systematic exposition of the biological and pharmacological underpinnings of these potential synergistic effects. The mechanistic evidence discussed herein is derived predominantly from *in vitro* assays, ex vivo models, and animal infection studies; while such evidence provides biological plausibility for combination regimens, it should not be conflated with clinically validated endpoint benefits (e.g., mortality reduction or attenuation of organ failure) addressed in Section 5. Furthermore, the distribution of mechanistic evidence varies across different macrolides, with agent-specific details noted in the respective subsections and summarized in **Table 3** and **Figure 3**. The ensuing text is organized according to direct and indirect mechanistic categories, which differ substantially in the strength of supportive evidence (as summarized in **Table 3**).

**Table 3:** Representative mechanistic studies, level of evidence, and translational limitations.

Direct/indirect mechanism	Representative studies	Study type	Mechanistic finding	Level of evidence	Clinical benefit	Key limitations
Complementary antibacterial targets	Lima et al., 2020; Vazquez-Laslop and Mankin, 2018	Molecular pharmacology	β-Lactams inhibit PBPs and peptidoglycan synthesis; macrolides inhibit 50S ribosomal translation.	A	C	Distinct targets do not establish synergistic killing or clinical benefit.
Cell-wall injury and increased macrolide penetration (hypothesized)	Vander Elst et al., 2025; Zhou et al., 2024	*In vitro*	In unconventional envelope-disruption models, cell-envelope disruption altered antibiotic susceptibility in Gram-positive experimental systems.	C	C	Does not demonstrate enhanced macrolide penetration with conventional β-lactam–macrolide combinations.
Efflux modulation (limited)	Sugimura et al., 2008	*In vitro*	In an *in vitro P. aeruginosa* study, subinhibitory azithromycin (~1.0 µg/mL; ~1/6 MIC) downregulated MexAB-OprM and altered susceptibility to aztreonam and carbenicillin.	B	C	Single experimental system; no evidence of patient benefit or class-wide efflux inhibition.
Suppression of bacterial repair and stress responses	Xu et al., 2025a	Animal model	In serial-passage and animal models, azithromycin repressed *rpoS* translation and delayed emergence of ceftazidime/avibactam resistance.	B	C	One drug pair and one pathogen; no patient-centered outcomes.
Biofilm modulation	Hoffmann et al., 2007	*In vitro*/animal model	In both *in vitro* systems and the Cftr−/− murine model of chronic *P. aeruginosa* infection, azithromycin was found to alter quorum-sensing signaling, alginate production, matrix composition, and susceptibility to host defenses.	B	C	Chronic infection model; not representative of acute CAP or standard combination therapy.
Host immunity	Giamarellos-Bourboulis et al., 2024	Human RCT	In a double-blind RCT in adults with CAP, clarithromycin altered TNF-α responses in LPS-stimulated PBMCs, circulating IL-10, and IL-8/IL-10 balance.	A	C	Single macrolide and regimen; immune mediation inferred from ex vivo analyses, not directly proven; trial not powered for mortality.
Quorum-sensing inhibition	Zeng et al., 2016; Molinari et al., 1993; Apostolaki et al., 2024	*In vitro*	In *in vitro P. aeruginosa* studies, subinhibitory macrolides suppressed las/rhl signaling and QS-regulated products (elastase, rhamnolipids, pyocyanin).	B	C	Mainly *in vitro* evidence at subinhibitory exposure; no causal link to benefit in acute combination therapy.

The Level of evidence column grades how well the mechanism itself is supported by experimental evidence. The Clinical benefit column grades whether the mechanism has been shown to contribute to clinical benefit. Both columns use the framework defined in Section 2 and do not imply the same certainty as outcome-level evidence in **Table 1**. For the Complementary antibacterial targets row, Level A reflects the well-established actions of each drug class rather than demonstrated synergistic killing or clinical benefit (Clinical benefit: C). For the Host immunity row, Level A reflects only the human RCT-derived cytokine findings; the macrophage, NETosis, dendritic-cell, and Th17 effects are preclinical or ex vivo and remain Level B (Clinical benefit: C).

**Figure 3:**
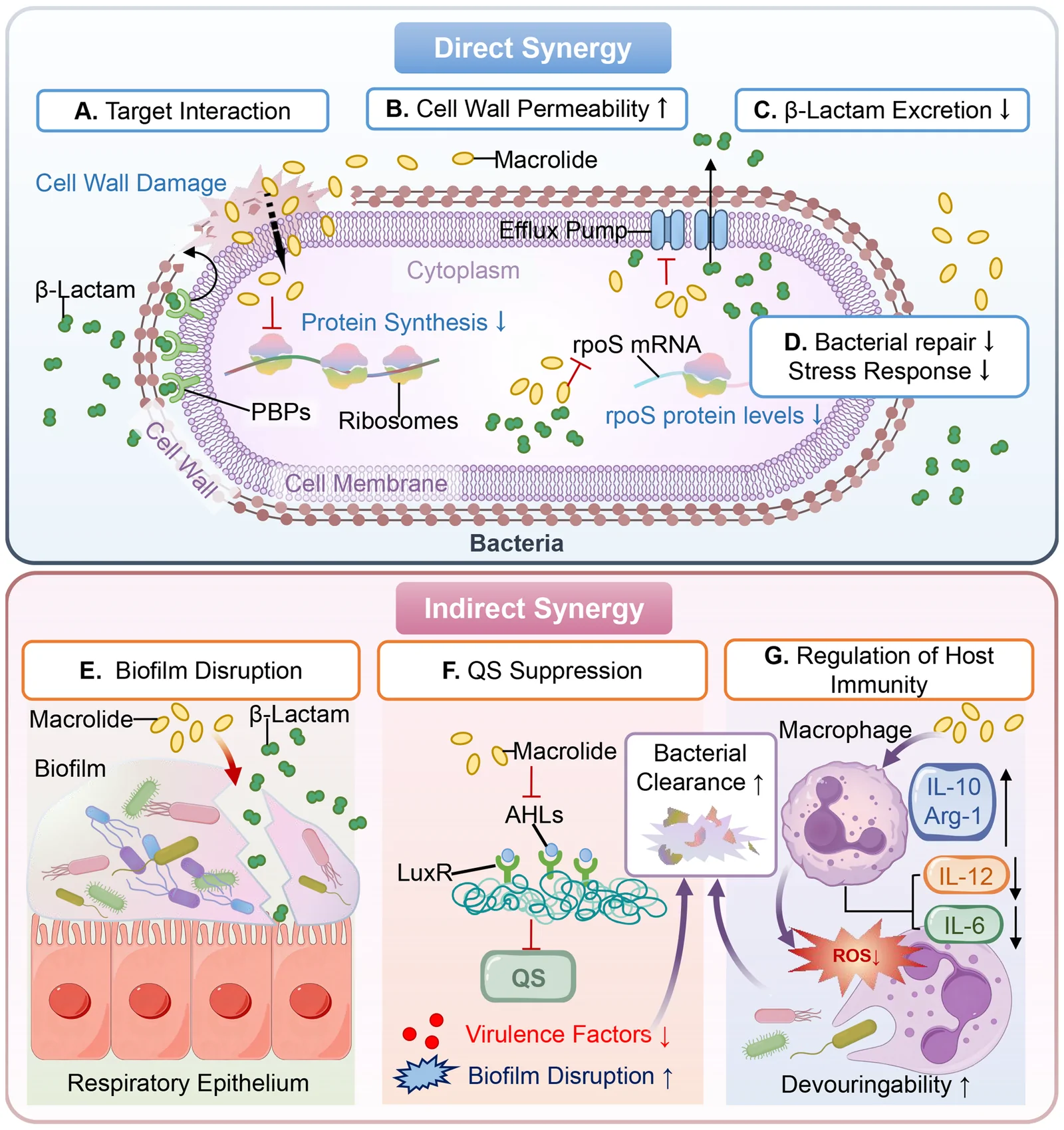
Hypothesized direct and indirect mechanisms of β-lactam-macrolide interactions and their evidential limits. **(A)** PBP inhibition and 50S ribosomal binding are established drug actions, but target complementarity does not prove synergy. **(B)** Increased macrolide accumulation after β-lactam-mediated envelope injury remains hypothetical for conventional clinical combinations. **(C)** Efflux modulation is supported only in selected experimental systems. **(D)** Azithromycin-mediated suppression of *rpoS* has been demonstrated in *P. aeruginosa* exposed to ceftazidime/avibactam but is not a class-wide mechanism. **(E)** Biofilm effects and **(F)** quorum-sensing effects are based mainly on preclinical, pathogen-specific studies. **(G)** Macrolides modulate host immune pathways, but a causal role in clinical benefit has not been established.

### 6.1 Hypothesized direct mechanisms of synergy and PK/PD complementarity

#### 6.1.1 Complementary antibacterial targets

β-Lactams and macrolides act on two well-defined targets: β-lactams inhibit PBPs and peptidoglycan synthesis, whereas macrolides bind the 50S ribosomal subunit and inhibit translation (Drago et al., 2011; Dinos, 2017; Vazquez-Laslop and Mankin, 2018; Lima et al., 2020; Svetlov et al., 2020; Turner et al., 2022; Pradhan et al., 2024; Taguchi et al., 2024). Simultaneous disruption of these essential processes is biologically plausible and may be complementary. Target complementarity alone, however, is not equivalent to synergistic killing. The direction and magnitude of the interaction depend on the pathogen, resistance phenotype, drug pair, concentration, order of administration, and infection environment (D'Agate et al., 2025; Gajic et al., 2025).

Beyond direct antibacterial synergy, macrolides may temper excessive inflammation. In a murine model of MRSA pneumonia, translation inhibition by a macrolide produced an adjunctive immunomodulatory effect and improved survival (Yamashita et al., 2019). That study did not test a conventional β-lactam–macrolide regimen and cannot directly support such treatment for MRSA. More broadly, combination therapy may be considered for resistant organisms when active monotherapy is inadequate, but only when each component has a defensible role.

Therefore, complementary target engagement is supported by established molecular pharmacology (Level A), but there is no direct evidence that concurrent target inhibition produces greater-than-additive killing in clinically relevant settings (Level C). The Level A assignment in **Table 3** therefore refers to the well-established actions of each drug class, not to the demonstration of synergistic killing.

#### 6.1.2 Cell-wall injury and the hypothesis of increased macrolide penetration

The hypothesis that β-lactam-mediated cell-wall damage facilitates macrolide entry is plausible but has not been demonstrated directly for conventional clinical combinations. Bacteriophage-derived endolysins cleave specific peptidoglycan bonds, disrupt the cell wall, and can increase macrolide entry and antibacterial activity (Vander Elst et al., 2025). Azalomycin F4a, an unconventional polyhydroxy macrolide analogue, interferes with peptidoglycan synthesis and substantially lowers the methicillin MIC in Gram-positive experimental systems (Zhou et al., 2024). These analogies show that disruption of the cell envelope can alter susceptibility, but they do not establish that standard β-lactams reproducibly increase intracellular accumulation of azithromycin, clarithromycin, or erythromycin.

Gram-negative organisms pose a greater barrier since their additional outer membrane, composed of lipopolysaccharide and porins, excludes many antibiotics. Effective penetration is often difficult. The clinical benefit of a β-lactam–macrolide combination against Gram-negative infection may therefore be limited (Lee et al., 2024; Mammeri et al., 2025; Su et al., 2025).

This mechanism remains hypothesized for conventional β-lactam-macrolide combinations and is not supported by direct evidence in human infection (Level C).

#### 6.1.3 Efflux modulation as a limited mechanism

In one study, subinhibitory azithromycin downregulated *mexAB-oprM* expression in *P. aeruginosa* and altered susceptibility to several agents (Sugimura et al., 2008). This pathogen-specific *in vitro* observation does not show that therapeutic macrolides act as clinically effective efflux-pump inhibitors. Macrolides are themselves often efflux substrates, and dedicated efflux-pump inhibitors represent a separate class of potentiators (Piddock, 2006; Blanco et al., 2016; Devi et al., 2025; Ojha et al., 2025).

At present, efflux modulation is best viewed as a probable but limited mechanism in selected experimental systems, not a general explanation for clinical benefit (Level B).

#### 6.1.4 Suppression of bacterial repair and stress responses

Translation inhibition could reduce synthesis of proteins required for stress responses and cell-envelope repair, rendering bacteria more vulnerable to β-lactam injury. The strongest specific evidence comes from azithromycin combined with ceftazidime/avibactam against *P. aeruginosa*, in which azithromycin suppressed *rpoS* translation and delayed resistance emergence in serial-passage and animal studies (Xu et al., 2025a). β-lactams can induce envelope, oxidative, and *rpoS*-associated stress responses (Gutierrez et al., 2013), while chaperone systems such as DnaK and HtpG help bacteria survive proteotoxic stress (Brand et al., 2025).

Direct evidence remains limited, however, that therapeutic macrolides broadly inhibit these pathways or improve clinical β-lactam killing through this route. This mechanism is therefore probable in defined experimental systems but drug- and pathogen-specific; its contribution to clinical benefit has not been demonstrated (Level B).

### 6.2 Indirect mechanisms of synergy

The indirect mechanisms of synergy discussed below are summarized in **Figure 3**.

#### 6.2.1 Macrolide modulation of biofilm structure and possible enhancement of β-lactam activity

Biofilms contribute to antibiotic evasion through the EPS matrix, which restricts drug diffusion and creates metabolic gradients that reduce β-lactam efficacy against slow-growing bacteria. At subinhibitory concentrations, macrolides can modify biofilm-associated processes: azithromycin suppresses QS signals and alginate production in *P. aeruginosa*, alters biofilm matrix composition and spatial organization, and inhibits biofilm formation in *P. aeruginosa* and *A. baumannii* (Hoffmann et al., 2007; Lees et al., 2020; Yamabe et al., 2020). These changes may reduce matrix density and improve penetration by antibiotics and host immune effectors.

Macrolide effects on biofilms involve reduced matrix production, lower virulence expression, and altered population behavior rather than direct killing of dormant cells. β-lactams, by contrast, act mainly on proliferating bacteria engaged in wall synthesis. By modifying the biofilm microenvironment, macrolides could expose some organisms to β-lactam activity. The magnitude of this effect depends on the organism, biofilm maturity, macrolide exposure, and the accompanying β-lactam.

The evidence derives mainly from *in vitro* biofilm experiments and animal models of chronic *P. aeruginosa* infection (Hoffmann et al., 2007; Lees et al., 2020; Yamabe et al., 2020). Clinical studies in chronic respiratory diseases have generally examined long-term macrolide effects on exacerbations or QS activity rather than directly testing biofilm clearance by acute combination therapy (Chalmers et al., 2019; Lees et al., 2020; Yamabe et al., 2020; Huang et al., 2022; Zahedi et al., 2025). Biofilm modulation therefore remains a probable preclinical mechanism; its contribution to clinical benefit in acute CAP has not been directly established (Level B).

#### 6.2.2 Modulation of host immunity

Beyond antibacterial action, macrolides can modify host immune responses and may complement β-lactam-mediated bacterial clearance. In the ACCESS trial, adjunctive clarithromycin altered the TNF-α response of LPS-stimulated PBMCs, circulating IL-10 concentrations, and the IL-8/IL-10 balance, suggesting modulation of infection-related immune dysregulation (Giamarellos-Bourboulis et al., 2024).

At the cellular level, macrolides affect multiple innate and adaptive immune compartments. They can dampen pro-inflammatory macrophage activation, promote an M2-like phenotype with enhanced phagocytosis, and reduce mediators including IL-6 and IL-12 (Amantea et al., 2016; Wang et al., 2018; Zhang et al., 2019; Pollock and Chalmers, 2021; Tarique et al., 2024). They may also promote neutrophil apoptosis and inflammatory resolution, though effects on oxidative burst, degranulation, and NETosis are less consistent (Abdelghaffar et al., 1996; Koch et al., 2000; Culić et al., 2002; Konstantinidis et al., 2016; Bystrzycka et al., 2017; Moges et al., 2018). In adaptive immunity, macrolides can modify dendritic-cell maturation and T-cell responses, including suppression of CD4^+^ T-cell proliferation and Th17 responses, possibly involving PI3K/Akt/mTOR signaling (Iwamoto et al., 2011; Lin et al., 2016; Weng et al., 2019; Pollock and Chalmers, 2021).

Taken together, these data suggest that macrolides may limit inflammatory injury while preserving pathogen clearance. This provides a host-directed rationale for combining them with β-lactams but does not prove that immune modulation mediates clinical benefit. Cytokine modulation is supported by patient-derived data (Level A), whereas enhanced macrophage phagocytosis, effects on NETosis and dendritic cells, and Th17 responses remain predominantly probable based on preclinical evidence (Level B).

#### 6.2.3 Quorum-sensing inhibition as a possible indirect mechanism

Quorum sensing (QS) regulates virulence-factor production, motility, adhesion and biofilm formation across bacterial species (Apostolaki et al., 2024; Dong et al., 2024; Liu et al., 2025; Touati et al., 2025; Ruan et al., 2026). At subinhibitory concentrations, azithromycin suppresses the las and rhl systems of *P. aeruginosa*, reducing signaling molecules and QS-regulated products such as elastase, rhamnolipids, and pyocyanin (Molinari et al., 1993; Zeng et al., 2016; Apostolaki et al., 2024; Leroy et al., 2024). In chronic *P. aeruginosa* lung-infection models, it also reduces alginate production and attenuates biofilm-associated phenotypes (Hoffmann et al., 2007). Erythromycin can similarly reduce α-toxin expression controlled by the agr system in *S. aureus* (Ohlsen et al., 1998), indicating that QS modulation may extend to Gram-positive organisms.

In a secondary analysis of a bronchiectasis trial, long-term erythromycin reduced *P. aeruginosa* QS activity (Burr et al., 2016). This provides some evidence of QS modulation in humans, but it was derived from chronic macrolide monotherapy rather than acute combination treatment for CAP.

QS inhibition is therefore best regarded as a probable mechanism at the preclinical level, with consistent *in vitro* and supportive animal data. Its clinical relevance—particularly whether it contributes meaningfully to the benefits of macrolide–β-lactam combination therapy in patients—has not been directly confirmed (Level B).

In summary, proposed mechanisms span inhibition of cell-wall synthesis and protein translation, modulation of bacterial stress responses, biofilm disruption, quorum-sensing interference, and immunomodulation. However, only a subset of these pathways has been convincingly linked to improved clinical outcomes, including enhanced bacterial clearance, attenuated inflammation, or better organ function.

While the fundamental pharmacology—β-lactam binding to PBPs and macrolide binding to the 50S ribosomal subunit—is well established, the translational relevance of most ancillary mechanisms remains incompletely defined. Preclinical observations of stress-response suppression, biofilm alteration, and quorum-sensing modulation are inherently context-dependent, varying with pathogen species, resistance genotype, drug concentration, and the infection microenvironment. Moreover, clinical benefits may derive independently from atypical pathogen coverage, appropriate treatment timing, or host-directed immunomodulation rather than from direct drug–drug synergy.

The hierarchy of importance among these mechanisms is likely to shift across pathogens, drug pairs, and infection niches, precluding a one-size-fits-all model. Well-designed translational studies integrating pharmacokinetic measurements, pathogen profiling, and mechanism-anchored biomarkers are needed to disentangle the relative contribution of each pathway.

## 7 Clinical challenges in combination therapy

### 7.1 Indications for combination therapy

At present, the delineation of appropriate clinical indications for β-lactam–macrolide combination therapy remains a major challenge. In routine practice, positive signals derived predominantly from observational studies are frequently overinterpreted, leading to the empirical extension of combination regimens beyond severe community-acquired pneumonia (CAP) to a broad array of infectious conditions, including invasive pneumococcal disease, biofilm-associated chronic infections, and methicillin-resistant *Staphylococcus aureus* (MRSA) pneumonia. However, such expanded applications lack robust high-level evidence. The underlying reasons for this predicament can be summarized as follows. First, high-quality randomized controlled trials have only confirmed an early clinical improvement benefit, whereas any mortality advantage has consistently been derived from confounded observational studies and indirect comparisons. Second, the mechanistic responses of different pathogens to macrolides are fundamentally heterogeneous (e.g., immunomodulation, atypical coverage, and antibiofilm effects are frequently conflated), and the biological rationale established in one pathogen or infection type cannot be directly extrapolated to others. Third, discrepancies in evidence grading among major guidelines (e.g., those of the ATS/IDSA versus the ERS/ESICM) further compound the uncertainty in clinical decision-making.

Currently, the only relatively well-supported scenario for combination therapy remains severe CAP or documented atypical pathogen infection, with the benefit primarily manifested as improved early clinical outcomes, while a mortality reduction has not been definitively established. For other infection types, reliable clinical evidence consistent with the pathogen-drug biological rationale is lacking.

Future research should prioritize: (1) stratifying patients by etiology and resistance-gene testing to identify subgroups likely to benefit; and (2) conducting rigorously designed RCTs in severe CAP populations, with mortality as a key primary endpoint, to provide a solid evidence base for defining precise indications. On this foundation, blind expansion of combination therapy based solely on mechanistic or indirect evidence should be avoided.

### 7.2 Dose optimization and PK/PD considerations

Complementary activity first requires both drugs to achieve effective exposure at the infection site over overlapping periods. β-Lactam activity is governed mainly by the proportion of the dosing interval during which the unbound concentration exceeds the minimum inhibitory concentration (fT>MIC). Critical illness frequently alters volume of distribution, protein binding, and renal clearance, making exposure under standard regimens unpredictable. Prolonged or continuous infusion can improve target attainment in severe infection, augmented renal clearance, or infection with a pathogen that has a higher MIC; therapeutic drug monitoring may be used when appropriate (Roggeveen et al., 2020; Goutelle et al., 2021).

Macrolide pharmacokinetics differ substantially from those of β-lactams. Azithromycin accumulates in tissues and cells, so plasma concentrations alone do not adequately reflect exposure in lung tissue or epithelial lining fluid. Activity against susceptible extracellular organisms is commonly related to AUC/MIC, whereas tissue distribution and intracellular concentrations are more important for intracellular or atypical pathogens such as *M. pneumoniae* and *Legionella species*. Prolonged tissue residence supports sustained action and shorter courses, but the subinhibitory tail during elimination may also increase selection pressure for resistance (Lebel, 1993). Extending treatment or increasing the dose will therefore not necessarily improve the outcome of combination therapy.

PK/PD considerations relevant to combination therapy are summarized in **Table 4**. The PK/PD properties of macrolides and β-lactams are distinct and potentially complementary. β-Lactams exhibit time-dependent killing, with efficacy primarily determined by the duration of time that free drug concentrations exceed the MIC (fT>MIC). They have limited tissue penetration and a minimal PAE (Goutelle et al., 2021). By contrast, macrolides demonstrate concentration- and time-dependent killing, with AUC/MIC and Cmax/MIC as key PD indices. They achieve high tissue concentrations and possess a prolonged PAE (Lebel, 1993).

**Table 4:** PK/PD considerations for β-lactam–macrolide combination therapy.

Component	PK/PD characteristics	Potential role	Practical implication
β-Lactam	Efficacy depends mainly on the fraction of the dosing interval during which the free drug concentration exceeds the MIC (fT>MIC); volume of distribution and clearance are often altered in critical illness	Sustained PBP occupancy and cell-wall injury	Adjust the dose according to renal function; in selected critically ill patients, consider prolonged infusion of the β-lactam and therapeutic drug monitoring
Azithromycin	Efficacy is related mainly to AUC/MIC and Cmax/MIC, indicating a need for adequate total exposure and peak concentration; it also has persistent intracellular and pulmonary exposure	Provides atypical-pathogen coverage and sustained tissue-level host-modulatory/antivirulence effects	Do not equate low plasma concentrations with absent tissue exposure; avoid unnecessarily prolonged dosing
Clarithromycin	Efficacy is also related to AUC/MIC and Cmax/MIC; it has an active 14-hydroxy metabolite and inhibits CYP3A4	Provides direct adjunctive-treatment evidence in CAP and immunomodulation	Review other co-administered drugs for potential CYP3A4-mediated interactions and monitor hepatic function; clarithromycin evidence cannot be extrapolated automatically to azithromycin
Erythromycin	Similar to azithromycin and clarithromycin, efficacy should consider AUC/MIC and Cmax/MIC; however, it has a short half-life, requires multiple daily dosing, and strongly inhibits CYP3A4	Experience mainly in chronic airway disease; limited contemporary evidence in acute CAP combination therapy	Poor gastrointestinal tolerability and interaction burden; not a routine first-line add-on for acute β-lactam–macrolide therapy
Combination	At the infection site, adequate β-lactam fT>MIC and adequate macrolide AUC/MIC, Cmax/MIC, and tissue exposure should be achieved concurrently	May provide complementary bacterial and host-directed effects	Start promptly when indicated in severe CAP; reassess at 48–72 h and de-escalate according to diagnostic findings

PK/PD, pharmacokinetics/pharmacodynamics; MIC, minimum inhibitory concentration; fT>MIC, fraction of the dosing interval during which the free drug concentration exceeds the MIC; AUC/MIC, area under the concentration–time curve relative to the MIC; Cmax/MIC, maximum drug concentration relative to the MIC; PBP, penicillin-binding protein; CYP3A4, cytochrome P450 3A4; CAP, community-acquired pneumonia; GI, gastrointestinal.

These complementary characteristics may contribute to clinical synergy (Level C). The extensive tissue penetration and prolonged PAE of macrolides may compensate for the limited tissue distribution, short half-life, and minimal PAE of β-lactams; conversely, the rapid bactericidal activity of β-lactams against dividing organisms may reduce bacterial burden and enhance macrolide efficacy against residual organisms. This pharmacological complementarity supports the use of combination therapy in severe CAP, with subsequent de-escalation to β-lactam monotherapy once clinical improvement is achieved and atypical pathogens are excluded.

Dose selection should account for age, body weight, hepatic and renal function, infection severity, and organ support (Goutelle et al., 2021). In critically ill patients, expanded volume of distribution, altered protein binding, and abnormal clearance can cause either inadequate β-lactam exposure or accumulation, so doses must be adjusted as physiology changes. In children, validated weight- or body-surface-area-based dosing should be used and adjusted to organ function and clinical response. Importantly, individualization involves not only dose selection but also whether the combination is indicated and how long it should be continued; prolonged or unnecessary exposure should be avoided.

### 7.3 Adverse effects, drug interactions, and monitoring

β-Lactams and macrolides have different adverse-effect profiles. With β-lactams, the main concerns are immediate or delayed hypersensitivity and, in patients with impaired renal function or high-dose exposure, accumulation and neurotoxicity. Macrolides commonly cause gastrointestinal symptoms and may also prolong the QT interval, impair liver function, and interact with other drugs. Clarithromycin and erythromycin are stronger CYP3A4 inhibitors and can increase exposure to susceptible co-medications (Westphal, 2000). Before prescribing a macrolide, clinicians should assess underlying cardiac disease, electrolyte abnormalities, and other QT-prolonging drugs (Collaborators, 2024; Wang et al., 2024; Kuzucu et al., 2025; Radkowski et al., 2025). Monitoring during treatment can be guided by symptoms, electrocardiographic findings, renal and hepatic function, and concomitant medications.

Antibiotic-allergy labels also shape regimen selection. Some recorded “β-lactam allergies” are gastrointestinal intolerance, infection-related rashes, or remote childhood reactions that have resolved. An inaccurate label can divert patients from first-line β-lactams and increase use of broader alternatives (Gilissen et al., 2021; Feldman et al., 2024; Kuzucu et al., 2025). A detailed history of timing, clinical features, and subsequent exposure, followed when appropriate by skin testing or a supervised drug challenge, can identify low-risk patients and remove erroneous labels (Kitsos et al., 2022). A macrolide should be selected because the infection requires its spectrum, not simply as a substitute for an unverified β-lactam allergy.

### 7.4 Antimicrobial stewardship, resistance, and ecological impact

Macrolide resistance mechanisms differentially affect the combination: *erm*-mediated methylation generally abolishes direct macrolide activity, whereas *mef*/*msr*-mediated efflux may produce lower-level resistance. For infections requiring atypical coverage, resistance to the macrolide component cannot be rescued by the β-lactam. Regimen selection must therefore reflect local resistance epidemiology (Collaborators, 2024).

Macrolide exposure can also select for resistance in colonizing microbiota and environmental reservoirs. While a short CAP course differs from mass repeated dosing, the ecological consequences—including gut resistome disruption and environmental contamination—warrant attention (Doan et al., 2020; Oldenburg et al., 2022; Cachetas et al., 2024).

The decision to initiate combination therapy should integrate disease severity, atypical-pathogen probability, and local resistance data. An antibiotic time-out at 48–72 hours, guided by clinical response and microbiological findings, should inform de-escalation to β-lactam monotherapy when atypical infection becomes unlikely (**Table 5**).

**Table 5:** Challenges in combination therapy and corresponding stewardship measures.

Challenge	Potential consequence	Stewardship measure
Macrolide resistance in the target pathogen	Loss of direct macrolide activity; possible treatment failure when atypical coverage is essential	Use local susceptibility data and rapid pathogen/resistance testing; choose an agent with demonstrated activity against the pathogen rather than expecting a β-lactam to restore macrolide susceptibility
Selection for resistance in colonizing microbiota	Expansion of *erm*/*mef* resistance genes in colonizing microbiota, altered resistome composition, and possible co-selection of resistance to other antimicrobials	Restrict indications and duration; avoid repeated courses with limited clinical benefit or unclear indication; monitor resistance trends
Environmental and One Health dissemination	Antibiotic residues and resistant bacteria in wastewater, livestock, and agriculture extend community resistance reservoirs	Pair clinical stewardship with environmental surveillance and One Health actions, including responsible veterinary use and wastewater management
Unnecessary dual coverage	More adverse events, *Clostridioides difficile* risk, drug interactions, and antibiotic days without added benefit	Reserve combination therapy for situations with a clear indication and avoid unnecessary dual coverage
Drug-specific toxicity	QT prolongation, gastrointestinal effects, hepatotoxicity, and CYP-mediated interactions	Assess baseline ECG/electrolytes and co-medications; select the macrolide according to patient factors and interaction profile
Diagnostic uncertainty	Broad empirical treatment may continue despite a viral diagnosis or identification of a specific pathogen	Obtain appropriate specimens and perform pathogen testing as early as possible; perform an antibiotic time-out at 48–72 h to decide whether to continue, adjust, or stop therapy
Cross-context generalization	Misapplying CAP evidence to pertussis, MRSA infection, or other bacterial infections	Restrict combination therapy to appropriate indications and avoid extrapolating these findings to other bacterial infections without supporting evidence

CAP, community-acquired pneumonia; CYP, cytochrome P450; ECG, electrocardiogram; MRSA, methicillin-resistant *Staphylococcus aureus*; CDI, *Clostridioides difficile* infection. *erm* and *mef* refer to macrolide-resistance genes.

Under a One Health perspective, human, animal, and environmental health are closely interconnected; addressing health challenges requires interdisciplinary and multisectoral collaboration.

## 8 Future research and clinical prospects

Future experimental and clinical studies should directly link defined drug exposure and pathogen-specific mechanisms to patient-centered outcomes. Mechanistic biomarkers should be specified in advance, negative interaction studies should be reported, and analyses should be stratified by severity, pathogen, individual macrolide, resistance phenotype, and timing of de-escalation. Exploratory adjuncts must be clearly separated from the evidence that supports current β-lactam–macrolide practice.

### 8.1 Development of new combination regimens

New combinations, including selected β-lactam–macrolide regimens, may help address resistant organisms and improve treatment, but their development requires a mechanistic understanding of the interaction (Lees et al., 2020; Giamarellos-Bourboulis et al., 2024). Studies of molecular targets and resistance pathways can guide rational pairing, improve clinical performance, and support evidence-based stewardship. A new regimen should begin with a defined clinical need rather than target complementarity alone. Pediatric reports have associated β-lactam–macrolide treatment, including amoxicillin-based combinations, with shorter hospital stays and fewer treatment failures in some groups, but this benefit appears confined to selected subgroups, such as school-aged children or those with suspected atypical pathogens (Williams et al., 2017; Al Saeedy et al., 2020).

Pharmacological interactions within a proposed regimen must also be assessed. Some pairs increase overall antibacterial activity, while others are additive or antagonistic, and drug–drug interactions may alter both efficacy and toxicity (Giamarellos-Bourboulis et al., 2021; Amabile-Cuevas, 2023). Continued work on mechanisms and resistance should inform future recommendations as bacterial susceptibility patterns evolve.

### 8.2 Personalized anti-infective treatment

The rise of antimicrobial resistance and the complexity of infectious diseases call for more individualized treatment. Pathogen genomics and pharmacokinetics are particularly relevant when β-lactams and macrolides are combined.

Recent reports show that dual β-lactam therapy can eradicate resistant *Mycobacterium abscessus* isolates, illustrating how treatment can be tailored to a specific resistance profile (Moguillansky et al., 2023). Pathogen genomic analysis can identify resistance mechanisms and guide selection of drugs capable of overcoming them. Whole-genome sequencing, for example, can detect strains that carry or co-express macrolide- and β-lactam-resistance determinants (Berbel et al., 2022). Such information may support combinations with a greater probability of success and a lower risk of selecting further resistance.

Pharmacokinetic factors-absorption, distribution, metabolism, and excretion-are equally important in patient-specific dosing. Altered absorption after bariatric surgery, for example, can substantially change the PK of oral antibiotics and require careful dose adjustment (Anvari et al., 2020). Pharmacogenomics may add another layer of personalization because variations in drug-metabolism genes can produce markedly different responses to the same agent. Polymorphisms affecting cytochrome P450 activity may alter macrolide metabolism and increase toxicity or reduce efficacy in some patients (Leekha et al., 2011). Where validated and clinically actionable, genetic testing could help identify the agent or dose best suited to an individual.

Real-time PK monitoring can provide direct information on drug exposure and allow prompt adjustment. This is particularly relevant in critical illness, where renal function, hepatic function, and comorbidities can all alter antibiotic pharmacokinetics (Roggeveen et al., 2020). Integrating pathogen genomics with the patient’s drug-metabolism profile may optimize multidrug therapy, reduce adverse effects, and ultimately improve outcomes.

### 8.3 Exploratory adjunctive technologies

As resistant and biofilm-associated infections become harder to treat, bacteriophages and magnetic-field-assisted approaches are being investigated as adjuncts. Phages can lyse specific bacterial hosts and, in some experimental models, alter surface structures, metabolic states, or resistance pathways in ways that restore antibiotic susceptibility (Tran et al., 2025). Phage–antibiotic combinations may reduce bacterial burden, delay expansion of resistant subpopulations, and lower the risk of failure with a single treatment (Palma and Qi, 2024). Their effects vary with the phage, bacterial strain, antibiotic class, resistance phenotype, and order of administration; a given pair may be synergistic, additive, or antagonistic.

Magnetic-field-assisted treatment generally uses magnetic carriers or alternating magnetic fields to improve drug delivery. Experimental systems suggest that magnetic nanomaterials can penetrate biofilms, alter the local matrix, and increase antibiotic accumulation at the infection site (Somawardana et al., 2024). Performance depends on the material, field strength, exposure time, and loaded antibiotic. A magnetic field alone should not be regarded as a generally effective antimicrobial potentiator.

Evidence for both approaches remains mainly *in vitro* or animal-based, and studies of their use with β-lactam–macrolide regimens are particularly sparse. Clinical translation must address phage host range, phage resistance, manufacturing and quality control, the biosafety of magnetic materials, and standardized delivery. These technologies currently offer research directions for refractory resistant or biofilm infections; they do not replace conventional treatment based on microbiology and susceptibility.

## 9 Limitations of this review

This is a narrative review. We did not use a preregistered protocol, an exhaustive two-reviewer search, or formal risk-of-bias assessment. Study selection may therefore be affected by publication and citation bias, particularly because positive synergy experiments are more likely to be reported than neutral or antagonistic combinations. We did not conduct a new meta-analysis.

The cited evidence is heterogeneous in species, strain, infection model, drug pair, dose, order of administration, exposure duration, and definition of synergy. Many mechanistic studies used sub-MIC macrolide concentrations, nonclinical analogues, or animal models that may not reproduce unbound drug exposure at human infection sites. Our “established/probable/hypothesized” categories are an interpretive framework, not a validated grading instrument.

The clinical evidence is further limited by confounding by indication, variable adherence to guideline-concordant care, inconsistent detection of atypical pathogens, and the combined analysis of severe and non-severe CAP. Findings for clarithromycin cannot be assumed to apply to azithromycin or erythromycin, and conclusions from CAP cannot be extended to pertussis, chronic airway disease, Gram-negative biofilm infection, or MRSA. These limitations constrain causal inference from molecular mechanisms to improved clinical outcomes.

## 10 Conclusions

The evidence for complementary and potentially synergistic effects of β-lactam–macrolide therapy is clinically important but heterogeneous, with the strongest signal in selected severe bacterial infections, particularly CAP. In some severe CAP populations, combination treatment has been associated with better pathogen control and lower mortality in observational analyses, but the mortality benefit remains uncertain because of population heterogeneity and residual confounding.

Several mechanisms could contribute: complementary antibacterial targets, disruption of cell-wall integrity, context-specific changes in efflux and bacterial stress responses, modulation of biofilms, and host immunoregulation. Their evidential support is uneven, and most cannot yet be linked causally to patient outcomes. Multiple points of attack may be useful against resistant organisms, but this rationale is not a substitute for pathogen-specific activity.

A PK/PD-based model can help align exposure of both agents at the infection site and guide individualized dosing while minimizing toxicity. This precision is particularly important as resistance increases and treatment options narrow.

Further work should define which mechanisms operate in specific drug–pathogen pairs and whether they mediate clinical benefit. Patient factors, including host genetics, microbiome composition, and comorbidity, may also influence the balance of benefit and harm.

Adjuncts such as phage therapy and magnetic-field-assisted delivery may eventually extend the activity of selected antibiotic combinations in complex or refractory infection, but they remain experimental.

β-Lactam–macrolide therapy is thus a biologically and clinically defensible option for selected severe bacterial infections, especially CAP. Its value lies not simply in combining two familiar drug classes, but in choosing an appropriate regimen for a defined syndrome, the likely pathogen, and the individual patient. New agents will remain important, but careful use of existing antibiotics is equally central to preserving effective treatment.

The most defensible conclusion is conditional: β-lactam–macrolide therapy is a guideline-supported empirical option for selected patients with severe CAP and may benefit certain high-risk phenotypes, but it is not universally synergistic. Most proposed molecular pathways are pathogen- and exposure-specific, and several remain hypothetical. Clinical use should integrate severity, microbiology, local resistance, drug-specific pharmacokinetics and safety, and timely de-escalation.
